# Polysiloxanes and Silanes with Various Functional Groups—New Compounds for Flax Fibers’ Modification

**DOI:** 10.3390/ma16103839

**Published:** 2023-05-19

**Authors:** Weronika Gieparda, Marcin Przybylak, Szymon Rojewski, Beata Doczekalska

**Affiliations:** 1 Institute of Natural Fibres & Medicinal Plants—National Research Institute, ul. Wojska Polskiego 71B, 60-630 Poznań, Poland; 2 Poznan Science and Technology Park, Adam Mickiewicz University Foundation, ul. Rubież 46, 61-612 Poznań, Poland; 3 Department of Chemical Wood Technology, Faculty of Forestry and Wood Technology, Poznań University of Life Sciences, ul. Wojska Polskiego 38/42, 60-637 Poznań, Poland

**Keywords:** natural fibers, modification, polysiloxane, silanization, flammability, thermal stability, scanning electron microscopy, FTIR

## Abstract

There is an increasing desire to use natural products that will be both effective and biodegradable. The aim of this work is to investigate the effect of modifying flax fibers with silicon compounds (silanes and polysiloxanes), as well as examining the effect of the mercerization process on their properties. Two types of polysiloxanes have been synthesized and confirmed by infrared spectroscopy (FTIR) and nuclear magnetic resonance spectroscopy (NMR). Scanning electron microscopy (SEM), FTIR, thermogravimetry analysis (TGA) and pyrolysis-combustion flow calorimetry (PCFC) tests of the fibers were performed. On the SEM pictures, flax fibers purified and covered with silanes were visible after treatment. FTIR analysis showed stable bonds between the fibers and the silicon compounds. Promising results of thermal stability were obtained. It was also found that modification had a positive effect on the flammability. The conducted research showed that the use of such modifications, in the context of using flax fibers for composites, can yield very good results.

## 1. Introduction

Natural fibers have been gaining popularity for a long time due to their biodegradability, and thus, their environmentally friendly nature. As a renewable raw material, natural fibers are used to produce recyclable ecological products, thereby reducing carbon dioxide emissions and the amount of waste generated by industrial processes. Flax fibers are used on a large scale by various industries, mainly for the production of textiles, paper and composites.

This work focused on the appropriate, effective two-step modification (mercerization followed by silanization) of flax fibers using specially synthesized polysiloxanes and commercially available silanes with different functional groups for their later use in composites. Such modification is necessary to obtain good adhesion in the composite. This has been a known problem for many years—the different polarity of the fibers and polymers—hydrophilic and hydrophobic, respectively—makes it difficult to combine them effectively [1]. To obtain a change in the polarity of natural fibers, some chemical modifications are used, such as: mercerization [2], acetylation [3], acrylation [4], benzoylation [5], silanization [6], peroxide treatment [7], isocyanate treatment [8,9] and enzymatic treatment [10].

Two methods of modification, mercerization and silanization, were used in this work. In the process of mercerization, the reactivity of cellulose is increased by breaking the hydrogen bonds due to of the action of NaOH. That allows for a better wetting of the fibers. Moreover, NaOH can transform cellulose-I to cellulose–II [11,12]. Alkaline treatment facilitates fiber fragmentation and disaggregation [13] and breaks fiber bundles into smaller pieces. This, therefore, results in a rough fiber surface, which improves the adhesion of the fibers to the polymer matrix [14]. There are many reports in the literature on optimal alkaline treatment conditions for use in natural fibers. These reports are very divergent. Mishra et al. [15] modified jute and sisal fibers for 2–72 h at room temperature with 5% NaOH. On the other hand, according to Symington et al. [16], the optimal modification is using a 2–10% NaOH solution for 10–30 min.

Meanwhile, silanization offers the possibility of using more complex reagents and introducing appropriate functional groups to achieve a “tailor-made” modification effect. It is possible to obtain additional benefits depending on the structure of the silicon compound used for modification. Natural fibers are composed mainly of cellulose, which is rich in hydroxyl groups. Formation of chemical bonds between these hydroxyl groups and the modifier offers the possibility of permanent fiber modification [17]. Organosilicon compounds should contain reactive groups in their structure, e.g., alkoxy or glycidyl groups, responsible for bonding with cellulose hydroxyl groups, and functional groups that would give the modified surface specific properties [18]. There are several literature reports on modification with fluorinated or long-chain chlorosilanes [19] and alkoxysilanes [20]. The silanization process begins with the hydrolysis of the alkoxy groups in the silicone compound, which are converted into more active silanols as a result of this process. Then, during condensation, the silanols condense to form a three-dimensional Si-O-Si (siloxane) structure. The hydrogen bonds with the surface of the fibers are formed by adsorption and then fixed by subsequent curing. Acid or base can be used to catalyze the hydrolysis and condensation reactions.

An interesting alternative to the above modifications is the use of polysiloxanes, which can be attached to the fibers but also cross-linked on their surface. Due to their linear structure, polysiloxanes can form a hydrophobic layer on the surface of fibers. Polysiloxanes are a class of materials that consist of a Si–O–Si framework and are characterized by good thermal and chemical stability and flexibility [21,22].

In our previous work, we focused on the modification of fibers in various forms (fiber, fabric, roving) using silanes with various functional groups [23]. We also used a two-step modification process (plasma and silanization) [24].

In this article, modifications of fibers by mercerization, silanization, and a combination of mercerization and silanization were carried out. The novelty here is certainly the use of specially designed polysiloxanes with different functional groups to maximize changes in the surface of the fibers and therefore their potential use in composites.

## 2. Materials and Methods

### 2.1. Materials

Flax fibers were prepared by IWNiRZ-PIB (Flax fibers). Other reagents used for modification were: isopropanol pure p.a. supplied by POCH^®^ (Gliwice, Poland), ethyl alcohol 96% pure p.a. supplied by POCH^®^ (Gliwice, Poland), N-(2-Aminoethyl)-3-aminopropyltrimethoxysilane provided by Unisil Sp. Z o. o. (Tarnów, Poland) and vinyltrimethoxysilane provided by Unisil Sp. z o. o. (Tarnów, Poland). Polysiloxanes were synthesized according to the method described in Section 2.2. Poly(dimethyl, hydrogen methyl)siloxane 50/25 was provided by Wacker (Munich, Germany), while 1-octene and other reagents and solvents for polysiloxanes synthesis were purchased from Merck (Darmstadt, Germany).

Formulas of the modifiers are shown in Table 1.

### 2.2. Synthesis of Polysiloxanes

**Scheme 1:**
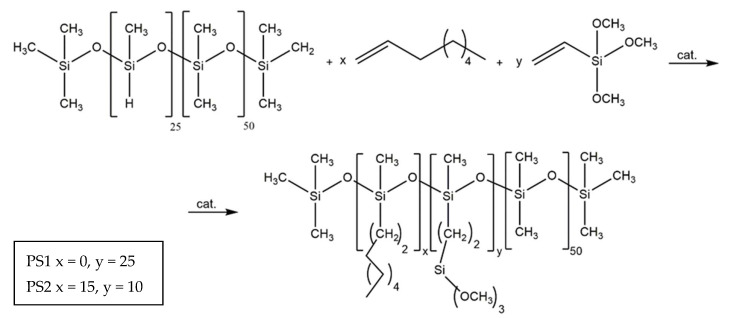
Synthesis of difunctional polysiloxanes.

Synthesis of difunctional polysiloxanes was carried out in two steps. The schematic reaction is presented on the Scheme 1 below.

Polysiloxanes containing long hydrocarbon chain groups and vinyltrimethoxysilane groups were synthesized by the hydrosilylation reaction of poly(dimethyl-co-hydromethyl)siloxane, 1-octene, and subsequently vinyltrimethoxysilane. The process was carried out in the presence of catalyst-Karstedt complex [Pt_2_{(CH_2_=CHSiMe_2_)_2_O}_3_]. At first, poly(dimethyl-co-hydromethyl)siloxane, 1-octene and the catalyst (5 × 10^−5^ mol Pt per mol Si-H) were put into a three-neck round-bottom flask with a thermometer, reflux condenser and magnetic bar at room temperature. The solution was then heated to appropriate temperature. Olefin conversion was monitored by FTIR analysis. Upon completion, the appropriate amount of the second olefin, vinyltrimethoxysilane, was added with a 10% excess. The solution was then kept at the set temperature for another hour. The reaction mixture was then cooled and the excess olefin was evaporated under reduced pressure. The structure of the obtained products was confirmed by NMR analysis. In the case of vinyltrimethoxysilane (PS1), the synthesis proceeded in the same way without 1-octene substitution.

### 2.3. Fibers Preparation and Modification

Flax fibers were dried at 50–55 °C for 24 h. Then, the material was disintegrated on a knife mill Retsch SM-200 (Haan, Germany) with a sieve that had a mesh size of 3 mm.

#### 2.3.1. Mercerization

The flax fibers were treated with 10% (*w*/*w*) NaOH aqueous solution for 10 min at room temperature. The NaOH/fibers weight ratio was 10:1. The fibers were then washed repeatedly in fresh distilled water until a neutral pH was obtained. Finally, all treated fibers were dried at 50 °C for 48 h.

#### 2.3.2. Modification with Silanes

After the mercerization process, one method of modifying the fibers was the reaction with two silanes with different properties—the more polar N-(2-aminoethyl)-3-aminopropyltrimethoxysilane and the less polar vinyl trimethoxysilane. An ethanol/water solution in the ratio of 6/4 (*v*/*v*) was prepared, to which the appropriate silane was added in the amount of 5% (*w*/*w*). Then, in the case of vinyl silane, the solution was acidified with acetic acid to pH 4.5. The hydrolysis process was carried out for 1 h. Fibers were then added and modification was carried out for 2 h at room temperature. The fibers were then placed in an oven set at 40 °C and dried. They were then cured for 10 min at 105 °C.

#### 2.3.3. Modifiaction with Polysiloxanes

An alternative method of fiber modification before and after the mercerization process (other than in the Section 2.3.2.) was silanization with two polysilooxanes with different functional groups and properties. The first was polisiloxane with alkoxy groups, and the second was difunctional polysilaxane with akloxy groups and alkyl chains. Modification with polysiloxanes was carried out in two ways: under the same conditions as modifications with silanes (ethanol/water solution in the ratio of 6/4 (*v*/*v*)) and in the isopropanol/water solution in the ratio of 19/1 (*v*/*v*) (samples marked by “ip”). The appropriate polysiloxane was added in the amount of 5% (*w*/*w*). Then, the solution was acidified with acetic acid to pH 4.5. The rest of the procedure was performed as in Section 2.3.2. A one-hour hydrolysis process was carried out. The fibers were then added, and a two-hour reaction was carried out at room temperature. Next, the fibers were placed in an oven set at 40 °C and dried. Curing was carried out at a temperature of 105 °C for 10 min.

### 2.4. Test Methods

#### 2.4.1. Fourier Transform Infrared Spectrometry (FTIR) Analysis

FTIR spectra of the polysiloxanes were taken on a BRUKER spectrometer, model Tensor 27 (Billerica, MA, USA), with a Specac Golden Gate single reflection diamond ATR accessory (Orpington, UK).

The analysis of FTIR spectroscopy of modified fibers was performed in KBr pellets using a BRUKER IFS 66v/S spectroscope (Billerica, MA, USA) in the mid-infrared range of 4000–400 cm^−1^ with a resolution of 2 cm^−1^.

#### 2.4.2. Nuclear Magnetic Resonance Spectroscopy (NMR)

Spectra of nuclear magnetic resonance ^1^H NMR (300 MHz), ^13^C NMR (75 MHz), ^29^Si NMR (59 MHz) were obtained on a Varian XL 300 spectrometer (Palo Alto, CA, USA) at room temperature using CDCl_3_ as a solvent.

#### 2.4.3. Thermal Stability Tests

Thermogravimetric study (TGA) was performed with TA Instruments, Analyser Q50 (New Castle, DE, USA). A 15 ± 1 mg fiber sample was heated to 700 °C at a heating rate of 10 °C·min^−1^ under a nitrogen atmosphere with a constant gas flow rate of 90 mL·min^−1^. The mass loss curve and the first derivative of TG (DTG) were determined.

#### 2.4.4. Flammability Tests

Flammability tests were performed using a pyrolytic combustion flow calorimeter (PCFC) by FTT (Grinstead, UK) for fiber samples weighing 5±1 mg. Testing was carried out in accordance with ASTM D7309-2007. The heating rate was 1 °C·s^−1^. The pyrolysis temperature range was 75–500 °C, and the combustion temperature was 900 °C. The flow was a mixture of oxygen and nitrogen gases at a ratio of 20:80 cm^3^·min^−1^. The maximum heat release rate (HRR_max_) was determined.

#### 2.4.5. Scanning Electron Microscopy

Microscopic test photos of longitudinal views of flax fibers were made with a Hitachi S-3400N scanning electron microscope (SEM) using a secondary electron detector SE in a high vacuum mode. Prior to the tests, the fibers were sprayed with a gold layer. The value of the accelerating voltage was 20 kV, and the working distance was 20 mm. Magnifications of 500× were selected.

## 3. Results

First, the obtained polysiloxanes were analyzed by FTIR and NMR methods. Subsequently, analyses of raw and modified flax fibers were carried out. The results were divided into two sections: the results of polysiloxanes synthesis and the results of flax fibers. For better understanding, Table 2 containing all samples can be found below:

### 3.1. Results of Polysiloxanes Synthesis

**Figure 1:**
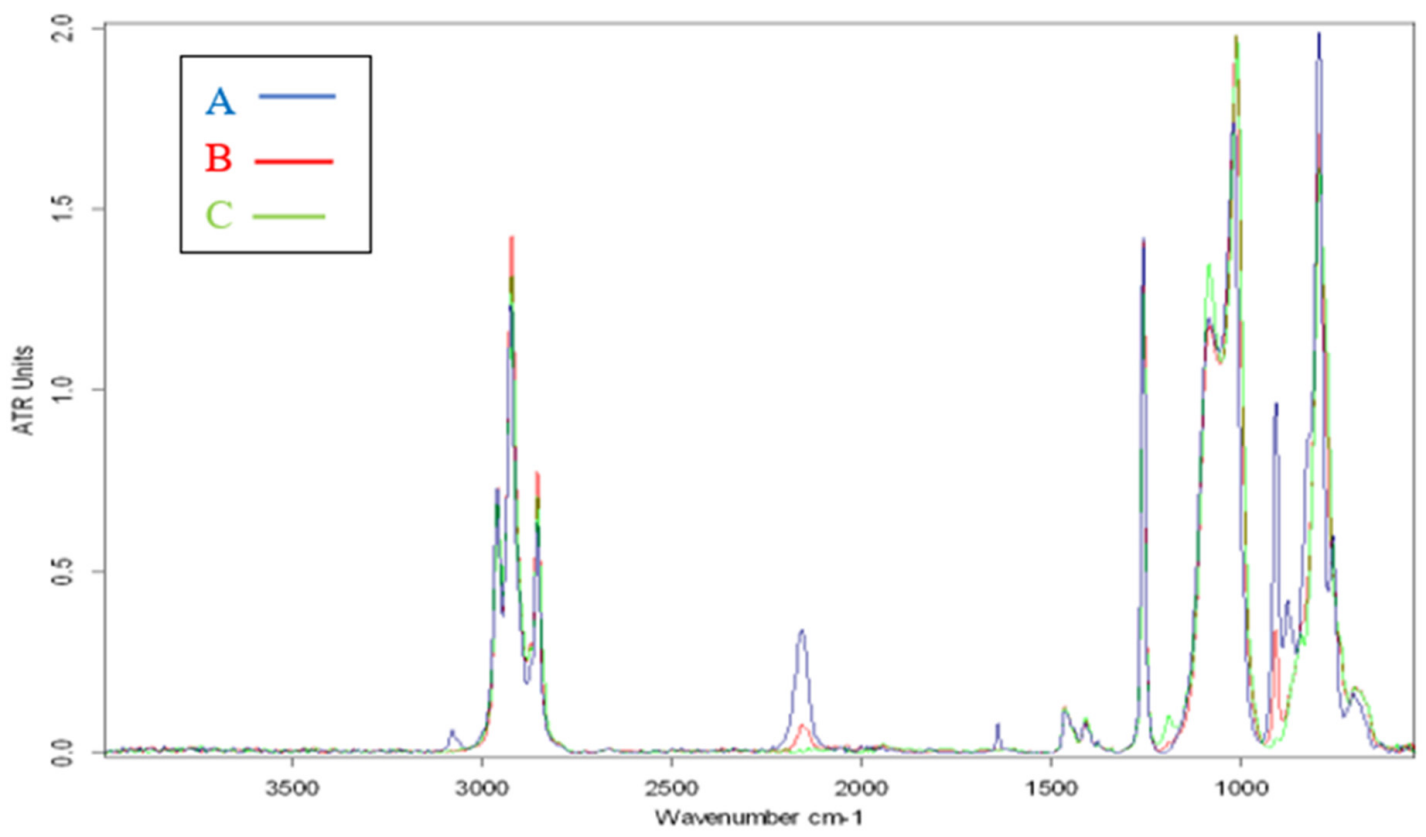
FTIR spectra of the starting mixture (A), after the introduction of hydrocarbon chains (B), and of the final product (C) for polysiloxane PS2.

Two polysiloxanes were synthesized via hydrosilylation with vinyltrimethoxysilane, and one of them additionally with 1-octene. Synthesis was carried out in the presence of Karstedt’s catalyst. The hydrosilylation process was monitored by FTIR spectroscopy. The intensity of the characteristic bands for the Si-H and CH=CH_2_ groups was analyzed. The process was conducted until the total disappearance of bands characteristic of unsaturated allyl groups at 3084 and 1650 cm^−1^, as well as bands characteristic of Si-H bond at 2193 and 855 cm^−1^ (Figure 1).

The successful synthesis was also confirmed by NMR spectroscopy. Appearance of specific signals was observed in the NMR spectra of the products. The polisiloxanes PS1 and PS2 were obtained with a yield of 93% and 92%, respectively:

(a) PS1 x = 0, y = 25 Yield = 93%
  ^1^H NMR (C_6_D_6_, 298 K, 300 MHz) (ppm): 0.14 (CH_3_); 0.64, 1.15 (CH_2_); 3.60 (OCH_3_);
  ^13^C NMR (C_6_D_6_, 298 K, 75.5 MHz) (ppm): 0.5 (CH_3_); 1.0 (CH_3_); 8.2 (CH_2_Si); 50.5 (OCH_3_);
  ^29^Si NMR (C_6_D_6_, 298 K, 59.6 MHz) (ppm): 7.2 (Si(CH_3_)_3_); *−*21.9 (Si(CH_3_)_2_); *−*22.7 (Si(CH_2_)CH_3_); *−*41.0 (Si(OCH_3_)_3_)
(b) PS2 x = 15, y = 10 Yield = 92%
  ^1^H NMR (C_6_D_6_, 298 K, 300 MHz) (ppm): 0,07 (Si(CH_3_)_3_); 0.1 (SiCH_3_); 0.55 (SiCH_2_); 0.91 (CH_2_CH_3_); 1.29 (CH_2_); 1.31 (CH_2_); 3.59 (54H, OCH_3_);
  ^13^C NMR (C_6_D_6_, 298 K, 75.5 MHz) (ppm): 0.5 (CH_3_); 1.0 (CH_3_); 8.2 (CH_2_Si); 14.1 (CH_3_); 17.5 (CH_2_Si); 22.7 (CH_2_); 23.0 (CH_2_); 29.3 (CH_2_); 29.4 (CH_2_); 31.9 (CH_2_); 33.4 (CH_2_); 50.5 (OCH_3_);
  ^29^Si NMR (C_6_D_6_, 298 K, 59.6 MHz) (ppm): 7.2 (Si(CH_3_)_3_); *−*22.0 (Si(CH_3_)_2_); *−*22.2 (Si(CH_2_)CH_3_); *−*41.5 (Si(OCH_3_)_3_).

### 3.2. Results of Flax Fibers

#### 3.2.1. Scanning Electron Microscopy Images of Flax Fibers

**Figure 2:**
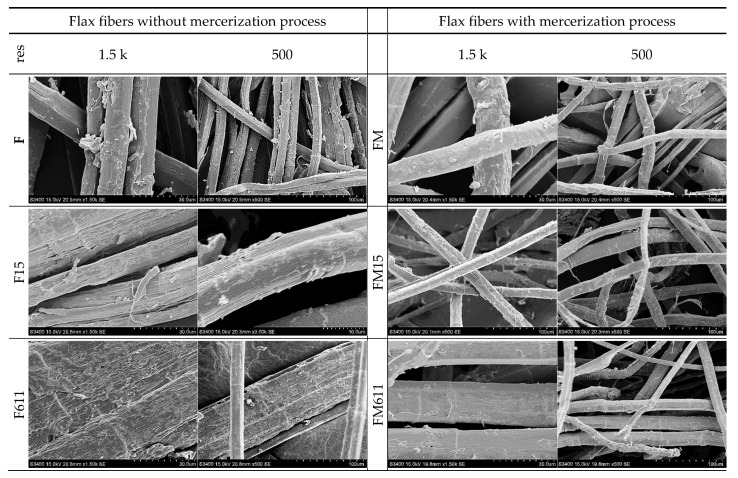

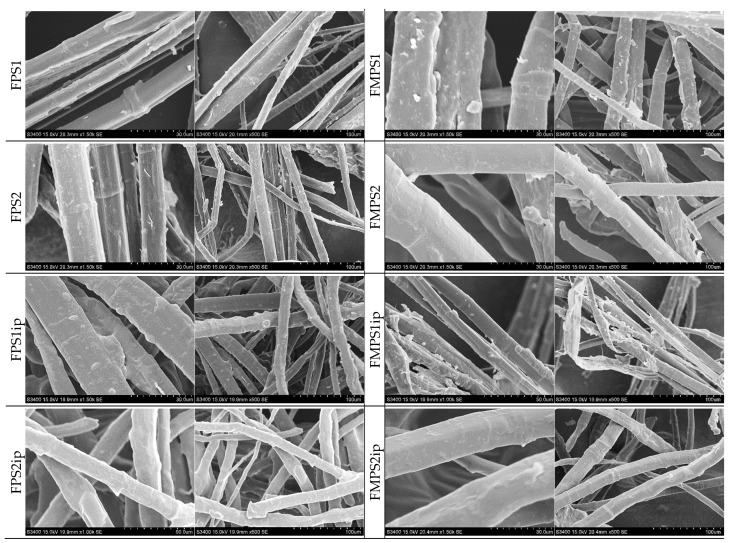
Scanning electron microscopy images of flax fibers without mercerization process and flax fibers with mercerization process in two resolutions: 500× and 1500×.

The effect of the modification and the presence of silanes/polysiloxanes on the surface of flax fibers was confirmed by SEM. Surface morphology of flax fibers before and after modifications were investigated. Figure 2 shows SEM images of unmodified and modified flax fibers. Longitudinal electron micrograph images of the fibers were taken in two magnifications: 500 and 1500 times.

Microscopic analysis of the surface morphology of the fibers is of great importance for characterizing the structural changes that have occurred after treatment. The main role of NaOH treatment is to clean the fiber of impurities and prepare the fiber for further processing. This is a widely used chemical process which removes noncellulosic components and part of the amorphous cellulose [25]. In turn, silane treatment is very helpful in removing lignin and hemicelluloses from natural fibers. In the photos of raw flax fibers, impurities on the fiber are clearly visible. After the mercerization process, the fibers are visibly cleaned. Aminosilane and vinylsilane modified fibers, both with and without prior mercerization, are also purified and covered with a thin layer of silanes. However, there is a clear difference in the photos of fibers modified with polysiloxanes. The fibers have been evenly covered with a layer of polysiloxanes, and their surface is smooth and clean. It was observed that the modifier covered the surface of the fibers in a relatively thick but smooth layer.

Many researchers have noticed that the fibers after NaOH treatment become purified and more susceptible to the action of silanes. In turn, modification with silane allows the fibers to be covered with a uniform layer, and as such, the fibers’ microscopic images show a smoothened surface on the fibers [26]. Puglia et al. [27] reported that NaOH can remove surface impurities from fibers while silane makes them smoother. SEM images from Liu et al. [28] showed that the surface morphology of treated corn stalk waste fibers was slightly rough and relatively clean after silane treatments.

Scanning electron microscopy is a great method for characterizing fibers and the effects of modifications on their texture. However, to confirm that, in addition to physical changes on the surface of the fibers, stable bonds between the fibers and the silane have been formed, it is worthwhile to carry out other analyses. The mere adsorption of silane to the fiber will not improve the adhesion between the silane and the polymer in the composite [29].

Accuracy of diameter measurement of natural fibers is very difficult to achieve because natural fibers are irregular in shape and thickness [30]. Mercerization is a process that “cleans” the fiber of waxes, pectins, etc., which naturally leads to a reduction in the diameter of the fibers. On the other hand, modification with silanes or polysiloxanes causes the modifier to bind to the fiber and form a layer covering the fiber, which can lead to an increase in the diameter of the fibers.

**Figure 3:**
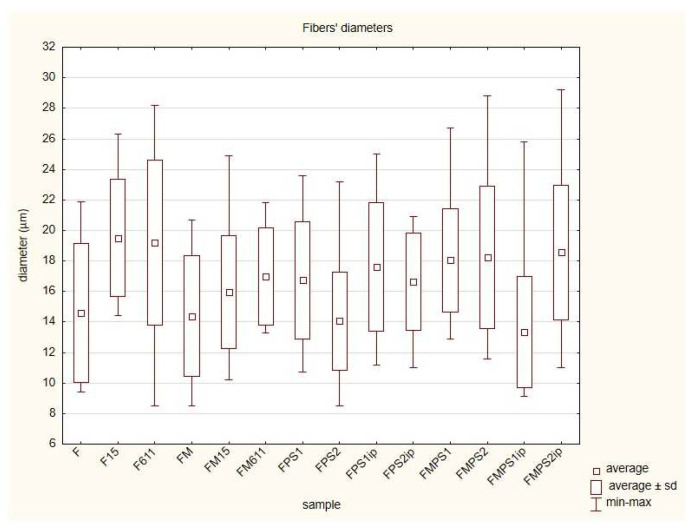
Fibers’ diameters before and after modifications, as well as standard deviation and average diameters.

The diameters were tested based on images from a scanning electron microscope. Despite large divergencies in the size of the fibers (Figure 3), it was observed that after mercerization, the average diameter of the fibers slightly decreases, while in the case of modification with silicon compounds, it increases. The largest increase in average diameter was observed in the case of aminosilane modification. Interestingly, with the mercerization and polysiloxane 1 modification, a decrease in the diameter of the fibers was observed.

#### 3.2.2. FTIR Tests

**Figure 4:**
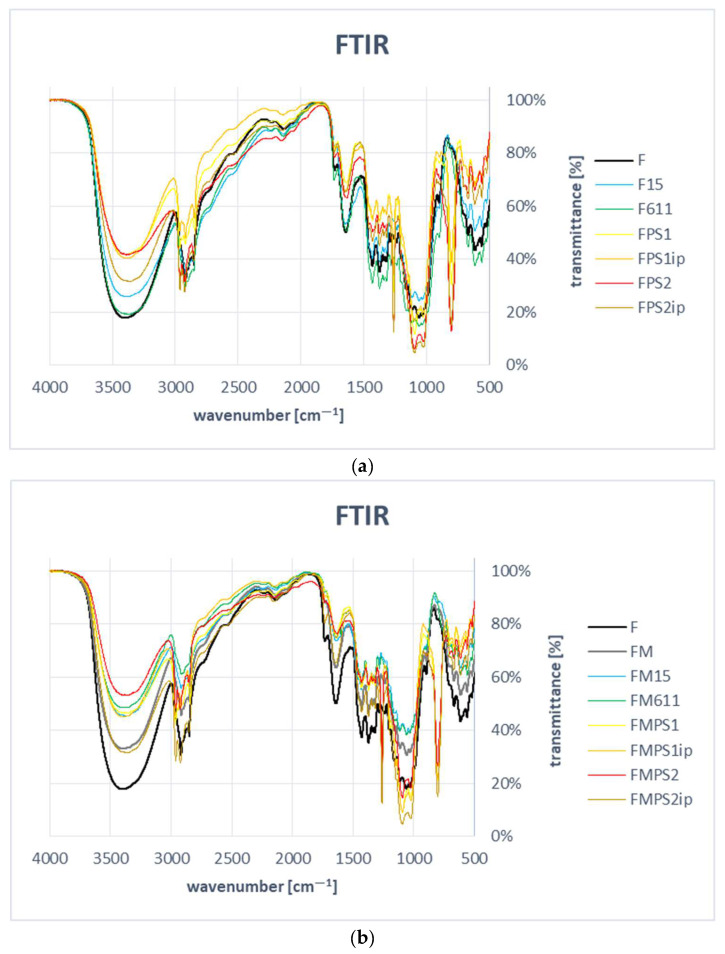
FTIR spectra (in the region of 4000–500 cm^−1^) of fiber samples: (**a**) one-step modification; (**b**) two-step modification.

Using FTIR spectroscopy, structural changes on the fiber surface after silane treatment were examined, which confirmed that the silicon compounds were chemically grafted onto the fiber surface. The following Figure 4a shows the IR spectra for samples modified in one step (silanization only) and Figure 4b shows the IR spectra for samples modified in two steps (mercerization and silanization).

Characteristic absorption bands for cellulose molecules appear in all tested samples [31,32,33]. The wide band, ranging from about 3000 cm^−1^ to 3500 cm^−1^, comes from the stretching vibrations of the O-H groups in the cellulose. In all cases, the band after modification is less intense, which indicates the occurrence of bonding between alkoxysilanes and the fibers.

The bands in the spectral region of 2800–3000 cm^−1^ assigned to C–H stretching vibration are characteristic of alkylene (–CH_2_–) and alkyl (CH_3_) groups, which were bound to fibers because of the modification with silicon compounds. The decrease in the intensity of these bands in some fiber samples may be caused by the reduction of the crystalline structure of cellulose. On the other hand, intensity of these bands increases because of the increased content of CH_2_ groups in the silanes and polysiloxanes. This band increases especially after fiber modification with polysiloxane with a long alkyl chain (PS2). Moreover, in the spectra of samples modified with both types of polysiloxanes, an additional band appeared at 2962 cm^−1^ that is ascribed to CH_3_ groups.

The vibration band visible at 1734 cm^−1^, resulting from the C=O stretching vibrations of the acetyl group in hemicellulose and aldehydes in lignin [34], disappears or is slightly reduced in the case of mercerized fibers. This is due to the degradation of hemicellulose and the dissolution of lignin during the alkali treatment of fibers.

An absorption band in the range 1630–1650 cm^−1^ originates from the stretching vibrations of the O-H group and correspond to absorbed water in crystalline cellulose [35]. Other characteristic bands resulting from vibrations in the cellulose molecule (CH_2_ bending vibrations) can be observed in all tested samples with wave number values of approx. 1430 cm^−1^ and 1370 cm^−1^.

In the spectra of fibers modified with aminosilane, a weak band at 1570 cm^−1^, characteristic of primary amino groups, is seen. Furthermore, the spectra of the samples modified with polysiloxanes contain bands at 801 and 1260 cm^−1^, originating from Si–O–Si symmetric stretching vibrations and the Si–O–C stretching vibration shoulder, respectively.

In the region of 1000–1200 cm^−1^, three characteristic bands appear for the cellulose molecule. At approx. 1160 cm^−1^, a band of the asymmetric C−O−C stretching vibrations in cellulose was observed. At approx. 1110 cm^−1^, a band of the C−OH skeletal vibration in cellulose was observed. At approx. 1050 cm^−1^, C−O−C pyranose ring skeletal vibrations were ascribed to cellulose. These bands are reduced in all tested samples after modification, and can be attributed to the reduction in the crystalline structure of the cellulose after treatment. The band expected at 1018 cm^−1^ (Si–O–Si) overlapped with this broad region that corresponds to the characteristic peaks of cellulose [21].

#### 3.2.3. Thermal Stability Tests of Flax Fibers

Natural fibers consist of hemicellulose, cellulose and lignin. Other ingredients, such as pectin and waxy substances, are not important in this context. The decomposition of natural fibers can be divided into four main stages (Yang et al. [36]). The first is the evaporation of moisture, followed by the decomposition of hemicellulose, and then the decomposition of cellulose and lignin. For a better understanding of the thermal properties of the fibers before and after silane treatment, information on the pyrolysis properties of these three main components is important.

**Figure 5:**
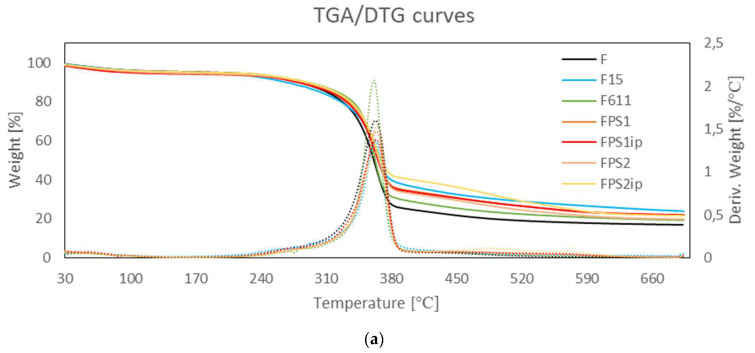

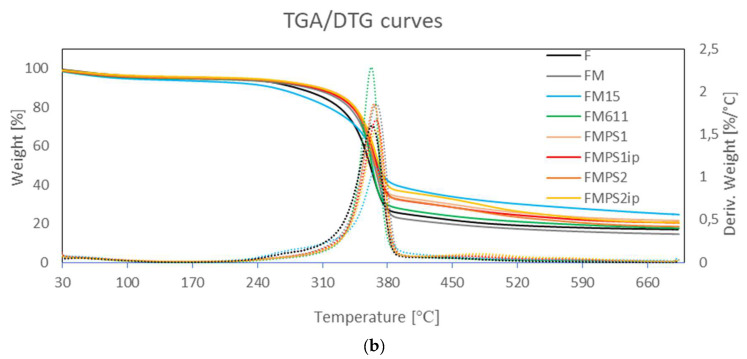
Thermogravimetric analysis of fiber samples: (**a**) flax fibers without mercerization process, (**b**) flax fibers with mercerization process. The solid lines represent the TGA curves, while the dotted lines represent the DTG curves.

The analysis of the TGA/DTG curves showed that the flax fiber decomposition process can be divided into three main stages (see Figure 5). In addition, the second stage can be divided into two substages, which are not clearly visible in all cases since these stages overlap, but it was decided to determine them for all fiber samples. These steps vary depending on the modification used.

All characteristic points and stages of decomposition as well as the first derivative peak temperature (DTG peak) were included in Table 3 below.

The first point to be considered is the temperature at which the fibers begin to decompose. That was assumed as an onset temperature (T_onset_). The temperature at this point was significantly higher for mercerized fibers, especially those additionally modified with polysiloxanes. The first stage of decomposition, reaching a temperature of about 150–170 °C, was the evaporation of water, and was characterized by a weight loss of 4.95–6.66%. Fibers modified with amine silane were characterized by the greatest weight loss in this area, but the differences between individual samples were small. The second stage—in which hemicellulose, amorphous cellulose and low-molecular compounds, i.e., waxes, pectin, etc., are decomposed—was separated for the temperature range of 176–227 °C to 305–320 °C, depending on the sample. It can be clearly seen that the beginning of this region shifts towards higher temperatures for mercerized fibers, except for fibers also modified with aminosilane. The weight loss in this step was 6.08–12.49, depending on the sample. The highest mass loss in this range is observed for samples modified with amine silane (both with and without prior mercerization). The third stage, which was the main stage of decomposition with the greatest mass loss (mainly cellulose degradation), was within the temperature range of 305–320 °C to 384–393 °C. At this stage, in the range 360–367 °C, depending on the type of fiber modification, the first derivative peak temperature (DTG peak) occurred. The peak of the first derivative indicates the point of greatest rate of change on the weight loss curve. This is also known as the inflection point. This point did not differ significantly between individual fiber samples. The mass of the tested samples decreased at the third stage by 41.48–63.9%. In this range, for samples modified with aminosilane, the lowest mass loss is observed (opposite to stage II). The fourth and last stage is the longest stage of decomposition, associated most probably with the slow degradation of lignin. The residue after the process ranged from 14.56–24.64%, depending on the sample. It can be noticed that the highest amounts (23.93% and 24.64%) of residual char belonged to the fibers modified with aminosilane.

It is clearly visible that the use of different silanes with different functional groups, as well as different polysiloxanes, significantly affect the thermal stability of the tested fibers. An increase in thermal stability was observed at the initial decomposition temperature, and then at the shift of the second stage of decomposition for mercerized samples. Modification of natural fibers with silicon compounds resulted in the formation of a silica layer on the fiber surface. This layer can create a protective barrier from the thermal radiation and stamp out the release of combustible gases. Mercerization facilitated the bonding of silicon compounds with flax fibers.

The flax fibers used in this research are very similar to another natural fiber—hemp fibers. They are also lignocellulosic fibers, and their modification occurs in a similar manner. Similar results can be expected with regard to thermal stability. In the literature, one can find that the use of the same two-step modification process on hemp fibers can lead to similar results. Rachini et al. [37] conducted research on the thermal stability of hemp fibers and the impact of mercerization and silanization on their thermal properties. He showed that both alkali treatment and silanization can improve the thermal decomposition of the hemp fibers.

#### 3.2.4. Microcalorimeter Tests of Flax Fibers

**Figure 6:**
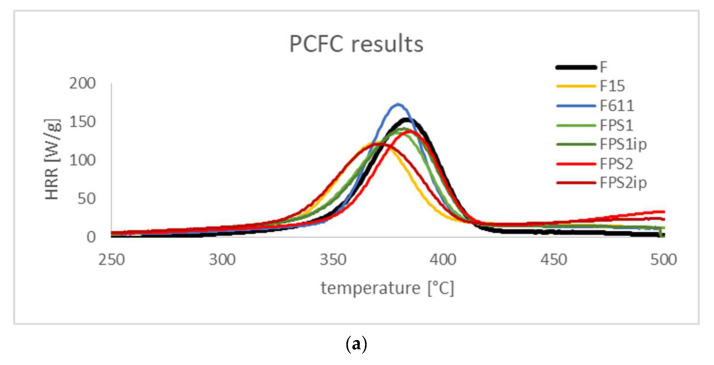

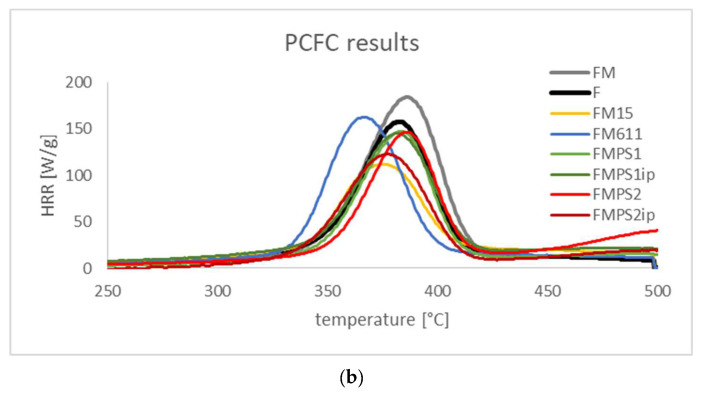
Microcalorimetric results of: (**a**) flax fibers without mercerization process, (**b**) flax fibers with mercerization process.

Figure 6 shows the HRR curves from the pyrolysis and combustion flow calorimeter (PCFC) test for samples of fibers modified only with silicon compounds (a), and first mercerized and then modified with silicon compounds (b).

The use of an amine silane usually results in a lower HRR peak due to the presence of amino groups in its structure. Interestingly, the use of a polysiloxane with a long alkyl chain also resulted in a decrease in HRR to similar values, especially when using isopropanol as a solvent in the silanization reaction. In these cases, there was an approx. 25% reduction in HRR_max_. For samples modified with polysiloxane without an alkyl chain, this reduction was slightly lower and amounted to about 10%. No significant differences in the height of the HRR curves were observed when comparing the mercerized samples (Figure 6b) and those that did not undergo this process (Figure 6a). Modification with vinyl silane caused an approx. 10% increase in HRR, which is a normal phenomenon for this type of modification [24].

The use of 95% isopropanol and only 5% water as a solvent for the polysiloxanes was preferable to the use of 60% ethanol and 40% of water as a solvent because polysiloxanes are very sensitive to water. If as much as 40% water was used, premature condensation of these compounds could occur, which in turn could hinder proper silanization.

There are few literature reports in which the flammability of natural fibers modified only with silicon compounds without the use of additional flame retardants is tested. Most often, researchers use phosphorous agents to reduce the flammability of natural fibers [38,39]. Various combinations of flame retardants are used in the literature to achieve a synergistic effect, further reducing the flammability of the fibers [40]. Unfortunately, when these compounds are used, the thermal stability of natural fibers is often reduced [41]. This phenomenon can be a serious problem, especially if the fibers are ultimately to be used in composites, the processing temperatures for which are often above 150 °C. In turn, the use of silicon compounds to modify natural fibers has a positive effect on thermal stability [42].

Alkali and silane/polysiloxane treatment used in our study showed that this type of modification can positively affect the flammability properties of the fibers without decreasing their thermal stability.

## 4. Conclusions

Promising results of modification of natural fibers with silicon compounds were obtained in the work:

- The successful synthesis of polysiloxanes was performed and confirmed by FTIR and NMR results;
- SEM photos of the fibers showed that they were cleaned because of mercerization and their diameter was reduced, and during silanization, were covered with a thin, uniform layer of silicon compounds;
- FTIR analysis showed that stable bonds between silanes or polysiloxanes and fibers were formed because of the modification;
- A two-step modification of flax fibers (Na OH treatment and then modification with polysiloxanes) increased the thermal stability of the fibers and increased the temperature of the initial fiber decomposition;
- Improvement of flammability properties was also obtained for the modifications with aminosilane and difunctional polysiloxane with a long alkyl chain.

In summary, it was observed in the conducted research that alkali treatment was important in the modification of flax fibers. The positive effect of the performed mercerization can be seen both in SEM images and in thermal stability studies. In addition, the functional groups of the silanes and polysiloxanes used also have a significant impact on the obtained test results. The use of an amino group in silane visibly increases its flammability properties, but also lowers thermal stability and leaves the largest residue after the TGA test. The incorporation of an alkyl chain into the polysiloxane structure allowed for an excellent improvement in flammability properties. Particular attention should be paid to the fact that the use of polysiloxanes as an alternative to silanes is promising in the context of using of fibers in composites and obtaining good adhesion with the polymer matrix, due to the uniform coverage of the fibers and the formation of permanent bonds with them.

## Figures and Tables

**Table 1:** Formulas of the modifiers used in the study.

Modifier	Abbrev.	Chemical Formula
N-(2-Aminoethyl)-3-aminopropyltrimethoxysilane	15	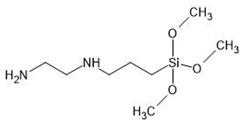
vinyltrimethoxysilane	611	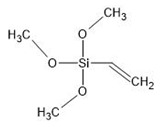
Polysiloxane with alkoxy groups	PS1	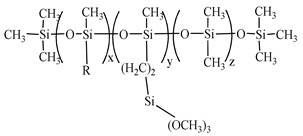 z = 50, y = 25, x = 0
Difunctional polysiloxane with alkoxy groups and alkyl chains	PS2	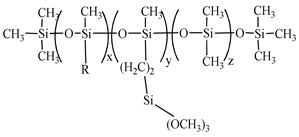 z = 50, y = 10, x = 15, R = (CH_2_)_7_CH_3_

**Table 2:** All samples prepared in the research.

Sample Code	Type of Modification
F	Untreated flax fiber
F15	Flax fiber + N-(2-Aminoethyl)-3-aminopropyltrimethoxysilane
F611	Flax fiber + vinyl trimethoxysilane
FPS1	Flax fiber + polysiloxane with alkoxy groups
FPS2	Flax fiber + difunctional polysiloxane with alkoxy groups and alkyl chains
FPS1ip	Flax fiber + polysiloxane with alkoxy groups (isopropanol as a solvent)
FPS2ip	Flax fiber + difunctional polysiloxane with alkoxy groups and alkyl chains (isopropanol as a solvent)
FM	Flax fiber + mercerization
FM15	Flax fiber + mercerization + N-(2-Aminoethyl)-3-aminopropyltrimethoxysilane
FM611	Flax fiber + mercerization + vinyl trimethoxysilane
FMPS1	Flax fiber + mercerization + polysiloxane with alkoxy groups
FMPS2	Flax fiber + mercerization + difunctional polysiloxane with alkoxy groups and alkyl chains
FMPS1ip	Flax fiber + mercerization + polysiloxane with alkoxy groups (isopropanol as a solvent)
FMPS2ip	Flax fiber + mercerization + difunctional polysiloxane with alkoxy groups and alkyl chains (isopropanol as a solvent)

**Table 3:** Thermal stability results of flax fibers.

Sample	T_onset_	DTG Peak	Stage Imass loss	Stage II		Stage III		Stage IV		Residue
				Start Temp.	Mass Loss	Start Temp.	Mass Loss	Start Temp.	Mass Loss	at 700 °C
	[°C]	[%·°C^−1^]	[%]	[°C]	[%]	[°C]	[%]	[°C]	[%]	[%]
F	333	363	5.29	213	10.21	313	58.73	389	8.79	16.98
F15	334	362	5.28	176	11.58	312	45.27	390	13.94	23.93
F611	336	360	5.71	202	9.3	321	54.43	384	11.2	19.36
FPS1	336	363	6.24	206	9.47	319	49.09	390	13.28	21.92
FPS1ip	333	363	5.87	195	9.22	314	50.19	390	12.72	22
FPS2	338	363	5.92	210	6.63	318	53.86	393	13.75	19.84
FPS2ip	333	362	4.95	188	7.27	305	46.91	391	19.23	21.64
FM	338	367	6.04	218	7.22	316	63.9	391	8.28	14.56
FM15	330	366	6.66	179	12.49	311	41.48	391	14.73	24.64
FM611	342	362	5.85	227	6.32	318	59.33	386	10.92	17.58
FMPS1	342	365	5.35	226	6.14	317	54.35	388	12.59	21.57
FMPS1ip	341	367	5.61	226	7.58	320	54.23	389	12.13	20.45
FMPS2	342	364	5.56	223	9.44	320	52.53	391	14.03	18.44
FMPS2ip	341	367	5	220	6.08	312	51.92	390	16.49	20.51

## Data Availability

Data sharing is not applicable.

## References

[1] Hasan A., Rabbi M.S., Billah M.M. (2022). Making the lignocellulosic fibers chemically compatible for composite: A comprehensive review. Clean. Mater..

[2] Ray D., Sarkar B.K., Rana A.K., Bose N.R. (2001). The mechanical properties of vinylester resin matrix composites reinforced with alkali-treated jute fibers. Compos. A Appl. Sci. Manuf..

[3] Tserki V., Zafeiropoulos N.E., Simon F., Panayiotou C. (2005). A study of the effect of acetylation and propionylation surface treatments on natural fibers. Compos. A Appl. Sci. Manuf..

[4] Li X., Tabil L.G., Panigrahi S. (2007). Chemical treatments of natural fiber for use in natural fiber-reinforced composites: A review. J. Polym. Environ..

[5] Nair K.M., Thomas S., Groeninckx G. (2001). Thermal and dynamic mechanical analysis of polystyrene composites reinforced with short sisal fibers. Compos. Sci. Technol..

[6] Pradeep S.A., Rodríguez L.J., Kousaalya A.B., Farahani S., Orrego C.E., Pilla S. (2022). Effect of silane-treated pine wood fiber (PWF) on thermal and mechanical properties of partially biobased composite foams. Compos. C Open Access.

[7] Fuqua M.A., Huo S., Ulven C.A. (2012). Natural fiber reinforced composites. Polym. Rev..

[8] Datta J., Kopczyńska P. (2015). Effect of kenaf fibre modification on morphology and mechanical properties of thermoplastic polyurethane materials. Ind. Crops Prod..

[9] Freddi G., Innocenti R., Arai T., Shiozaki H., Tsukada M. (2003). Physical properties of wool fibers modified with isocyanate compounds. J. Appl. Polym. Sci..

[10] De Prez J., Van Vuure A.W., Ivens J., Aerts G., Van De Voorde I. (2019). Effect of enzymatic treatment of flax on fineness of fibers and mechanical performance of composites. Compos. A Appl. Sci. Manuf..

[11] Doczekalska B., Borysiak S. (2008). WAXS studies of Salix viminalis wood susceptibility to alkali treatment. Fibres Text. East. Eur..

[12] Doczekalska B., Zborowska M. (2010). Wood chemical composition of selected fast growing species treated with NaOH. Part I: Structural substances. Wood Res..

[13] Iannace S., Ali R., Nicolais L. (2001). Effect of processing conditions on dimensions of sisal fibers in thermoplastic biodegradable composites. J. Appl. Polym. Sci..

[14] Ali A., Shaker K., Nawab Y., Jabbar M., Hussain T., Militky J., Baheti V. (2018). Hydrophobic treatment of natural fibers and their composites—A review. J. Ind. Text..

[15] Mishra S., Misra M., Tripathy S.S., Nayak S.K., Mohanty A.K. (2001). Graft copolymerization of acrylonitrile on chemically modified sisal fibers. Macromol. Mater. Eng..

[16] Symington M.C., Banks W.M., West O.D., Pethrick R.A. (2009). Tensile testing of cellulose based natural fibers for structural composite applications. J. Compos. Mater..

[17] Dan Y., Popowski Y., Buzhor M., Menashe E., Rachmani O. (2020). Amir, Covalent surface modification of cellulose-based textiles for oil–water separation applications. Ind. Eng. Chem..

[18] Xie Y., Hill C.A.S., Xiao Z., Militz H., Mai C. (2010). Silane coupling agents used for natural fiber/polymer composites: A review. Compos. A Appl. Sci. Manuf..

[19] Xue C.H., Jia S.T., Zhang J., Tian L.Q. (2009). Superhydrophobic surfaces on cotton textiles by complex coating of silica nanoparticles and hydrophobization. Thin Solid Films.

[20] Sun S., Qiu Y. (2012). Influence of moisture on wettability and sizing properties of raw cotton yarns treated with He/O_2_ atmospheric pressure plasma jet. Surf. Coat. Technol..

[21] Przybylak M., Maciejewski H., Dutkiewicz A., Dąbek I., Nowicki M. (2016). Fabrication of superhydrophobic cotton fabrics by a simple chemical modification. Cellulose.

[22] Przybylak M., Maciejewski H., Dutkiewicz A., Walentowska J., Foksowicz-Flaczyk J. (2018). Development of multifunctional cotton fabrics using difunctional polysiloxanes. Cellulose.

[23] Gieparda W., Rojewski S., Wüstenhagen S., Kicinska-Jakubowska A., Krombholz A. (2021). Chemical modification of natural fibers to epoxy laminate for lightweight constructions. Compos. A Appl. Sci. Manuf..

[24] Gieparda W., Rojewski S., Różańska W. (2021). Effectiveness of Silanization and Plasma Treatment in the Improvement of Selected Flax Fibers’ Properties. Materials.

[25] Fisher T., Hajaligol M., Waymack B., Kellogg D. (2002). Pyrolysis behavior and kinetics of biomass derived materials. J. Anal. Appl. Pyrol..

[26] Zhou F., Cheng G., Jiang B. (2014). Effect of silane treatment on microstructure of sisal fibers. Appl. Surf. Sci..

[27] Puglia D., Monti M., Santulli C., Sarasini F., De Rosa I.M., Kenny J.M. (2013). Effect of alkali and silane treatments on mechanical and thermal behavior of Phormium tenax fibers. Fibers Polym..

[28] Liu Y., Lv X., Bao J., Xie J., Tang X., Che J., Ma Y., Tong J. (2019). Characterization of silane treated and untreated natural cellulosic fiber from corn stalk waste as potential reinforcement in polymer composites. Carbohydr. Polym..

[29] Belgacem M.N., Gandini A. (2005). The surface modification of cellulose fibers for use as reinforcing elements in composite materials. Compos. Interfaces.

[30] Kabir M., Wang H., Lau K., Cardona F. (2013). Tensile properties of chemically treated hemp fibers as reinforcement for composites. Compos. B Eng..

[31] Szymanska-Chargot M., Chylinska M., Kruk B., Zdunek A. (2015). Combining FTIR spectroscopy and multivariate analysis for qualitative and quantitative analysis of the cell wall composition changes during apples development. Carbohydr. Polym..

[32] Liu X., Renard C.M.G.C., Bureau S., Le Bourvellec C. (2021). Revisiting the Contribution of ATR-FTIR Spectroscopy to Characterize Plant Cell Wall Polysaccharides. Carbohydr. Polym..

[33] Anu;Kumar A., Rapoport A., Kunze G., Kumar S., Singh D., Singh B. (2020). Multifarious pretreatment strategies for the lignocellulosic substrates for the generation of renewable and sustainable biofuels: A review. Renew. Energy.

[34] Sun X.F., Xu F., Sun R.C., Fowler P., Baird M.S. (2005). Characteristics of degraded cellulose obtained from steam-exploded wheat straw. Carbohydr. Res..

[35] Seki Y. (2009). Innovative multifunctional siloxane treatment of jute fiber surface and its effect on the mechanical properties of jute/thermoset composites. Mater. Sci. Eng. A.

[36] Yang H., Yan R., Chen H., Lee D.H., Zheng C. (2007). Characteristics of Hemicellulose, Cellulose and Lignin Pyrolysis. Fuel.

[37] Rachini A., Le Troedec M., Peyratout C., Smith A. (2009). Comparison of the Thermal Degradation of Natural, Alkali-Treated and Silane-Treated Hemp Fibers under Air and an Inert Atmosphere. J. Appl. Polym. Sci..

[38] Dorez G., Otazaghine B., Taguet A., Ferry L., Lopez-Cuesta J.M. (2014). Use of Py-GC/MS and PCFC to characterize the surface modification of flax fibers. J. Anal. Appl. Pyrol..

[39] Sonnier R., Otazaghine B., Viretto A., Apolinario G., Ienny P. (2015). Improving the flame retardancy of flax fabrics by radiation grafting of phosphorus compounds. Eur. Polym. J..

[40] Bocz K., Szolnoki B., Marosi A., Tábi T., Wladyka-Przybylak M., Marosi G. (2014). Flax fiber reinforced PLA/TPS biocomposites flame retarded with multifunctional additive system. Polym. Degrad. Stab..

[41] Bocz K., Szolnoki B., Wladyka-Przybylak M., Bujnowicz K., Harakály G., Bodzay B., Zimonyi E., Toldy A., Marosi G. (2013). Flame retardancy of biocomposites based on thermoplastic starch. Polimery.

[42] Arbelaiz A., Fernandez B., Ramos J.A., Mondragon I. (2006). Thermal and crystallization studies of short flax fiber reinforced polypropylene matrix composites: Effect of treatments. Thermochim. Acta.

